# Strategies for the Transformation of Waste Cooking Oils into High-Value Products: A Critical Review

**DOI:** 10.3390/polym17030368

**Published:** 2025-01-29

**Authors:** Valentina Beghetto

**Affiliations:** 1 Department of Molecular Sciences and Nanosystems, University Ca’ Foscari of Venice, Via Torino 155, 30172 Mestre, Italy; beghetto@unive.it; Tel.: +39-041-2348928; 2 Crossing S.r.l., Viale della Repubblica 193/b, 31100 Treviso, Italy; 3 Consorzio Interuniversitario per le Reattività Chimiche e La Catalisi (CIRCC), Via C. Ulpiani 27, 70126 Bari, Italy

**Keywords:** waste cooking oil, recycling, polymer synthesis, circular economy

## Abstract

Waste cooking oils (WCOs) are generated globally from households, the hospitality industry, and other sectors. Presently, WCOs are mainly employed as feedstock for biodiesel and energy production, strongly depending on the availability of WCOs, which are often imported from other countries. The objective of this review is to give an overall comprehensive panorama of the impacts, regulations, and restrictions affecting WCOs, and their possible uses for producing high-value products, such as bio lubricants, bio surfactants, polymer additives, road and construction additives, and bio solvents. Interestingly, many reviews are reported in the literature that address the use of WCOs, but a comprehensive review of the topic is missing. Published studies, industry reports, and regulatory documents were examined to identify trends, challenges, production statistics, environmental impacts, current regulations, and uses for high-value polymer production. The data collected show that WCOs hold immense potential as renewable resources for sustainable industrial applications that are in line with global carbon neutrality goals and circular economy principles. However, achieving this shift requires addressing regulatory gaps, enhancing collection systems, and optimizing conversion technologies. This comprehensive review underlines the need for collaborative efforts among policymakers, industry stakeholders, and researchers to maximize the potential of WCOs and contribute to sustainable development.

## 1. Introduction

Demand for and consumption of vegetable oils has significantly increased in the last 15 years, increasing from 83 Miot in 2000, to over 217 Miot in 2023, 167 Miot of which were employed for biodiesel production, while the remaining part was used primarily by the food, feed, and pharma industries [1,2].

**Figure 1:**
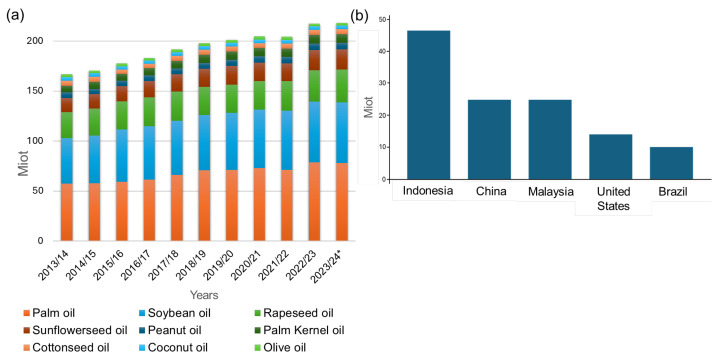
(**a**) Sources of Vegetable oils and (**b**) main geographical areas of production (Miot/year).

Globally, palm and soybean oils dominate the market with shares of 40.0% and 29.0%, respectively, while other vegetable oils are produced in smaller quantities, and include sunflower (9.7%), rape (6.3%), peanut (4.1%), corn (2.1%), coconut (1.9%), olive (1.8%), and sesame oil (1.1%) [2] (Figure 1a). Asia satisfies over 54.0% of worldwide vegetable oil demand; in particular, Indonesia is the world’s greatest producer, followed by China, Malaysia, the USA, and Brazil (Figure 1b) [3,4,5,6,7,8,9]. Although biodiesel may be considered an attractive alternative for the partial substitution of fossil-based fuels, edible oils are an essential source of nutrition, accounting for approximately 10.0% of the global average caloric food supply, second in importance only to cereals [10].

Additionally, it has been demonstrated that a correlation exists between the rise in biodiesel consumption in the USA and the increasing number of palm oil plantations in Southeast Asia, consequently increasing deforestation and global warming [11]. Thus, high production costs, and threats to the food and feed value chains, together with socio-environmental impacts, have made the use of edible oils as feedstock for biodiesel less appealing over time [12,13,14].

Alternative valuable feedstocks for biodiesel production are known, such as waste cooking oils (WCOs), fats, and grease, together with more recent technologies, starting from algae [1,15,16].

The term WCOs refers to exhausted cooking oils coming from the hospitality and industrial sectors, which are no longer suitable for human consumption [13]. If disposed of in drains or sewers, WCOs form products that linger in the environment for many years, generating odors and problems in wastewater treatment plants, and polluting soil and water courses, causing highly negative environmental impacts [17,18,19]. Additionally, WCOs may contain intermediate products, formed by oxidation or microbial degradation, which are toxic to microorganisms, algae, plankton, mussels, and amphipods [20,21], but also to humans, causing serious illnesses such as dyspepsia, stomach-ache, diarrhea, and gastric cancer [22]. The reuse and recycling of WCOs therefore open new frontiers, reducing the disposal costs and environmental impacts derived from them [23].

Presently, WCOs are mainly employed at the industrial level to produce biofuels and energy [24,25,26,27,28,29,30,31,32,33,34,35]; yet, in consideration of the EU requirements to reach carbon neutrality by 2050, the use of WCOs and, in general, of by-products and waste as fuels should be disfavored compared to their valorization for producing high-value chemicals [15,36,37]. In fact, the need to recover and recycle by-products or end-of-life products, reducing primary resource consumption and CO_2_ emissions, is becoming increasingly urgent, leading to a change from a linear economy based on a “take-make-discard” model, to a circular economy based on green chemistry and eco-design [38,39,40,41].

It should be further considered that the use of WCOs as feedstock for biorefineries continues to face a very critical moment in the EU. The dramatic decline in biodiesel prices, largely attributed to competition from Chinese companies, led to notable shifts in 2024, so that Chevron decided to shut down its biodiesel facility in Germany, BP reduced its sustainable aviation fuel and renewable diesel plans in Europe, and Shell stopped the construction of a major biodiesel plant in Rotterdam [42]. The contraction of the biodiesel market is an opportunity to boost the use of WCOs to produce high-added-value polymeric products at an industrial level, implementing innovative technologies and consequently supporting EU market competitiveness.

Alternative applications of WCOs include the production of bio lubricants [31,43,44,45], bio solvents [46], animal feed [47,48,49,50], asphalt additives [51,52], composite materials [53,54], polymers [55,56], fine chemicals [57,58,59], and non-aqueous gas sorbent devices [60,61], among others.

Within this panorama, the present review intends to give a general overview of the impacts, regulations, and restrictions affecting WCOs, and their possible uses, other than for fuel and energy, for producing high-value polymeric materials. The objective of this review is to give an overall comprehensive panorama. Data published in the last ten years in peer review papers have been subdivided according to the final application described. Interestingly, many reviews have been reported in the literature that address specific topics on the use of WCOs for the preparation of a specific class of polymer, but a general comprehensive review of this topic is missing.

## 2. Methods

Systematic research of the existing literature was carried out to analyze the current status of impacts, regulations, and restrictions affecting WCOs; the possible uses, other than fuel and energy, of WCO production, recycling, and upcycling technologies to produce high-value polymeric materials; and the impacts derived from these. A combination of different keywords were used in the search, such as “waste cooking oil”, “used cooking oil”, “bio lubricant”, “bio surfactant”, “bio solvent”, “polyurethane”, “bio polymer”, “non isocyanate polymer”, “acrylic polymer”, “alkyd ester”, “epoxy resin”, “asphalt/construction additive, rejuvenating, anti-aging”, “circular economy”, “EU directives”. Specifically, one or a combination of two keywords were chosen for the selection of the papers, and only English language papers published in peer review journals were included in the analysis.

The research papers included in the review were collected through Web of Science, Scopus, Google Scholar, ScienceDirect, and ResearchGate, and were published between 2014 and 2024. To find the most updated data and regulations, we also searched the websites of relevant organizations, such as the European commission portal [62], the European Law portal [63], the European Alliance of News Agency [64], the European Environment Agency (EEA) [65], the European Chemicals Agency (ECHA) [66], Statista [67], and Eurostat [68]. Once relevant sources were identified, references were reviewed for additional relevant information. This review focuses on the reuse of WCOs to produce polymeric materials, while papers concerning the use of WCOs for fuel and energy are not reported.

## 3. WCO Production and Market

Although much information is available in the literature on the production of vegetable oil worldwide (see Figure 1a,b), it is rather difficult to find accurate data quantifying the amounts of WCOs that are produced and available for valorization. Teixeira and coworkers reported that about 320 Kg of WCOs are produced for every ton of cooking oil used, with a variable valorization rate (between 75% and 23%), according to the geographical area [18]. A rather worrying fact that emerges from the analysis by Teixeira is that out of 23 states evaluated, over 85% had no specific legislation regarding WCO management. These data were also confirmed by a more recent paper by Zhao and coworkers, which indicated a general static trend over the recent years in the adoption of more sustainable management strategies for WCOs, and indicated that the main alternative remains inadequate disposal in the environment [69].

**Figure 2:**
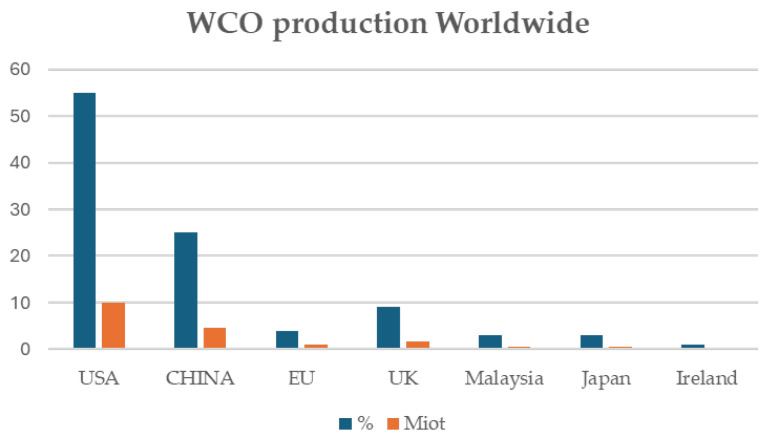
Geographical distribution of WCO production worldwide.

In the USA, according to the Environmental Protection Agency, nearly 11.4 Miot of WCOs are collected from restaurants yearly, only small percentages of which are adequately managed [70], while production estimates are of about 15.0 Miot of WCOs [71]. Even the virtuous EU has a long way to go, considering that according to estimations, the pro-capita production of WCOs in the EU is 8L/year, which, considering a population of around 500 million people, corresponds to a yearly production of WCOs of 4.0 Miot. This value, which is four times higher than the currently collected amount, clearly evidences the gap between WCO production and collection [44]. Another way to estimate the annual production of WCOs in the EU is by considering the region’s yearly consumption of vegetable oils, which amounts to 24.0 million tons (Miot). With 32% of this consumption being discarded, the annual production of WCOs in the EU could potentially reach around 7.7 million tons [18]. This also accounts for the great difference between vegetable oil consumption (220.0 Miot) and the amount of WCOs collected worldwide (about 15.0–18.0 Miot) [71], distributed geographically as reported in Figure 2.

Presently, WCOs are mainly employed by the industry as feedstock for biodiesel and energy production [72,73,74,75,76,77,78,79,80]. In fact, it is well known that WCOs can be used to produce fatty acid methyl esters (FAMEs) through alkali-catalyzed transesterification, and used as biodiesel [34,54,81,82]. Other processes for the valorization of WCOs are known, such as cracking or hydrocracking, pyrolysis, and gasification processes to produce biofuels [76]. Additionally, energy production by WCO combustion has been reported in the literature, under different conditions, and with WCOs having different viscosity and acidity [83,84].

It should nevertheless be considered that although the use of WCOs can reverse the noncompetitive price of biodiesel produced from virgin biomass and reduce negative environmental impacts [85,86], the use of WCOs as a primary feedstock for second-generation biodiesel production depends on their large-scale availability, and since local food waste is often insufficient, the importation of biodiesel or WCOs from other countries is required [87,88,89]. As evidenced by various LCA studies, the importation of WCOs is neither a sustainable practice to guarantee energy supply, nor does it contribute to reducing gas emissions [72,90]. In this concern, the recently approved EU renewable energy directive (Directive (EU) 2023/2413) states that “the share of biofuels and bioliquids, as well as of biomass fuels consumed in transport, where produced from food and feed crops, shall be no more than one percentage point higher than the share of such fuels in the final consumption of energy in the transport sector in 2020 in that Member State, with a maximum of 7% of final consumption of energy in the transport sector in that Member State” [91]. Within this panorama, the alternative use of WCOs for high-value products should be privileged as an alternative to their use as biodiesel. Thus, in this review, no further discussion on the use of WCOs for biodiesel production is reported.

## 4. Regulations and Restrictions Affecting WCOs

As far as the EU is concerned, starting from the 1970s, with the waste oil directive 75/439/EEC, the European Commission committed to collecting used oils, limiting environmental hazards, and promoting recovery and recycling technologies [44,92].

During the last decade, many regulatory updates have been implemented at the EU level for the classification, storage, recovery, or disposal of waste oils from hospitality, household, and other industrial compartments. Amending Directive 2008/98/EC and European Commission (EC) Decision 2000/532/EC, EC Decision 2014/955/EU of 18 December 2014 [93] reports a harmonized list of wastes, which is continuously updated based on new knowledge and research studies. It should nevertheless be stressed that the inclusion of materials in this list does not necessarily imply that the material is waste under all circumstances. A material is waste only when the definition of waste in Article 1(a) of Directive 75/442/EEC is met [94].

Different wastes are defined by a six-digit code (Directive 2014/955/EU) [93], where the first two digits identify the manufacturing compartment generating the waste, the second two, processing operations, and the last ones a specific material. This implies that waste may be identified by different EWC codes according to the manufacturing process that generates it, and vice versa, specific production units may need to classify their waste according to several codes. As regards WCOs, they are classified as 200125, where 20 corresponds to “municipal wastes (household waste and similar commercial, industrial, and institutional wastes)”, 01 to “separately collected fractions”, and 25 to “edible oil and fat”. Edible oils are also found as mixtures with grease coming from wastewater treatment plants, and are identified by EWC 190809, while if coming from agriculture, horticulture, aquaculture, forestry, hunting and fishing, food preparation, and processing wastes, the EWC is 0203.

Additionally, WCOs classified under EWC 200125 are not hazardous to human health, but their inadequate disposal makes them potentially harmful to the environment. For this reason, Art. 1 of Directive 2008/98/EC lays down the “measures to protect the environment and human health by preventing or reducing the adverse impacts of waste mismanagement and improving the efficiency for reuse”, requiring Member States to adopt waste management plans [95]. As in similar cases, the need to promote the development of a unified EU policy program, while preserving Member State sovereignty, in order to adopt independent approaches to waste management, recycling, and disposal, inevitably creates logistical barriers across borders [36].

Considering that in 2018, many Member States still had not yet developed the necessary waste management infrastructure, EU Directive 2018/851 was issued, amending EU Directive 2008/98/EC to “prevent the creation of structural overcapacities for the treatment of residual waste” and “improve the efficiency of resource use and ensuring that waste is valued as a resource, can contribute to reducing the Union’s dependence on the import of raw materials and facilitate the transition to more sustainable material management and to a circular economy model”(Directive (EU) 2018/851) [96]. This directive sets out the EU legislative framework pushing towards the achievement of the EU Action Plan and the Sustainable Development Goals of the Agenda 2030 to achieve carbon neutrality by 2050. It is of fundamental importance that a congruent and unified legislative framework is adopted within the EU to develop and promote near-zero waste processes [31,43,45,97].

As far as the US is concerned, back in October 1997, the Environmental Protection Agency (EPA) rejected a petition from several agricultural trade organizations seeking permission for facilities storing vegetable oils or animal fats to use less-stringent response methods for spill planning under the Facility Response Plan (FRP) rule (40 CFR 112.20-21; 1 July 1994) [98]. Subsequently, in 2000, the EPA introduced a revised FRP rule that clarified its applicability to facilities managing, storing, or transporting large quantities of animal fats and vegetable oils, particularly those transferring significant volumes of oil over water or storing at least one million gallons of oil. The updated rule aligned with the Edible Oil Regulatory Reform Act, by distinguishing animal fats and vegetable oils from other oil types based on their properties and environmental effects [99]. The revised rule introduced a specific methodology for calculating worst-case discharge planning volumes for animal fats and vegetable oils, using similar principles as those for petroleum oils, but incorporating tailored factors for estimating recovery needs on water and onshore. Separate regulatory sections were created for animal fats and vegetable oils, while maintaining the same response planning scenarios (small, medium, and worst-case discharge) from the original rule. New definitions for animal fats and vegetable oils were also added, along with a classification system dividing oils into Groups A, B, and C based on their specific gravity.

In 2002, the SPCC rule (40 CFR Part 112) was revised to address the Edible Oil Regulatory Reform Act’s requirements, dividing the rule into subparts [100]. Subpart A outlined general requirements for all facilities, Subpart B addressed petroleum and non-petroleum oils (excluding animal fats and vegetable oils), and Subpart C covered requirements for animal fats, greases, fish and marine mammal oils, and vegetable oils derived from seeds, nuts, fruits, and kernels. The revised rule also emphasized the significant environmental impact of oils on soil components. To advance sustainable processes aligned with the Circular Economy Action Plan, the adoption of a unified legislative framework is crucial, enabling the upcycling of waste and fostering near-zero waste processes [31,44,45].

In this connection, the EU End of Waste directive 2008/98 is a fundamental milestone, determining the necessary criteria to transform waste into a valuable secondary primary material [95,98]. Directive 2008/98/EC defines “recovery” as “any operation the principal result of which is waste serving a useful purpose by replacing other materials which would otherwise have been used to fulfil a particular function, or waste being prepared to fulfil that function, in the plant or in the wider economy” [98]. Additionally, “recycling” is defined as “any recovery operation by which waste materials are reprocessed into products, materials or substances whether for the original or other purposes not including energy … fuels or for backfilling operations”. Specifically, requirements for “End of waste status” are that a waste shall cease to be waste if the following conditions are met: (i) it has been demonstrated that “the substance is commonly used for specific purposes”; (ii) “a market or demand exists for such a substance or object”; (iii) “the substance fulfils the technical requirements for the specific purposes and meets the existing legislation and standards applicable to products” and (iv) “the use of the substance will not lead to overall adverse environmental or human health impacts”.

## 5. Environmental Impacts of WCOs

The environmental impacts derived from the inadequate disposal of WCOs are numerous, and derive from their accidental or deliberate release, causing environmental and biological alterations. WCOs’ highest environmental impacts occur when they are discharged into water bodies or indirectly enter them after being spilled into sewer systems [101,102,103]. WCOs are immiscible with water and generate a thin film, reducing oxygenation, limiting light filtration, and affecting the life of aquatic plants and living beings. It is estimated that 1 kg of used vegetable oil can be evenly distributed to cover an area of 1000 m^2^ [18]. Similarly, WCOs tend to form a hydrophobic film around earth particles and plant roots, creating a barrier to water and, consequently, precluding the intake of nutrients. Moreover, WCOs that penetrate the ground may reach drinking water wells, which become unsuitable for human consumption [102]. For this reason, it is essential to take appropriate precautions to protect groundwater and wells but also surface waters that may be in contact with the deeper water reservoirs. Moreover, like synthetic oils and fossil-based products, WCOs can cover the surfaces of living organisms (for example birds and aquatic animals), forming an insulating film which decreases their ability to breathe, exchange heat, and, in some cases, move.

The great risks associated with improper disposal of these substances has led to the development of a different regulation, the establishment of Consortiums devoted to the collection of WCOs, even if they are currently mandatory only for industrial activities, while the management of WCOs produced by citizens, defined as “urban waste”, is in the charge of municipalities.

## 6. WCO Composition and Pretreatments

In general, WCOs are mainly composed of 95% triglycerides with aliphatic chains ranging from 16 to 18 carbon atoms [104], derived from palm, soybean, canola, sunflower, peanut, cottonseed, coconut, olive, and corn oils [3] (Figure 1a).

**Figure 3:**
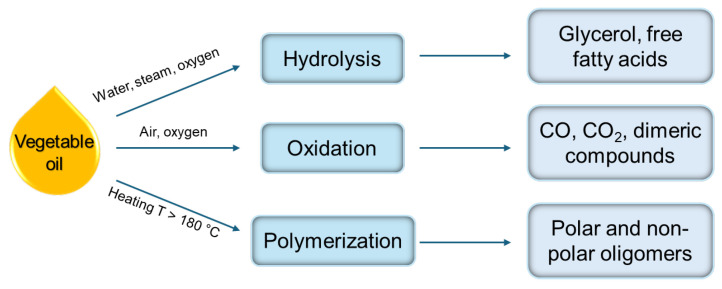
Chemical degradation reactions of vegetable oil.

WCOs are mostly produced from food frying at temperatures between 150 °C and 200 °C, and cannot be reused for food processing [55,105,106]. In fact, during use, vegetable oils undergo three main chemical reactions, i.e., hydrolysis, oxidation, and polymerization, which alter their physical chemical characteristics [3,48,104] (Figure 3). Hydrolysis occurs in the presence of moisture in food, resulting in the formation of glycerol and free fatty acids (FFAs), which cause color changes and the production of smoke. Oxidation reactions naturally occur when the double bonds in vegetable oil interact with atmospheric oxygen, leading to the formation of highly reactive hydroperoxides. Additionally, polymerization reactions take place between unsaturated fatty acyl groups, forming dimeric or oligomeric triacyl glycerides [48,55]. Additionally, thermal cracking of unsaturated fatty acids, followed by oxidation at high temperatures, may also promote polymerization reactions, forming a variety of toxic compounds such as heterocyclics [107].

WCOs differ [25,27] in color, viscosity, density, pH, flash point, number of unsaturated bonds, chemical composition, and toxic components, because of chemical degradation [13,59,108,109,110]. Data reported in the literature clearly highlight changes in the pH (from 5.34 to 7.38), kinematic viscosity (from 28.744 mm^2^/s to 68.568 mm^2^/s), and molecular weight of WCOs produced from different vegetable oils and under different cooking conditions (Table 1) [110,111]. For this reason, many different WCO pretreatment processes prior to recycling, such as filtration, extraction, and distillation to eliminate water and impurities, have been reported in the literature [26,112].

Additionally, the recycling of WCOs depends on their acidity, iodine value, and number of conjugated dienes or trienes, which influence their recyclability [55]. For example, it is evident that highly acidic WCOs need to be neutralized before base-catalyzed transesterification [27]. Further pretreatments may be necessary to meet specific standards, such as EN 14214 for biodiesel [113], while specific standards related to the volatile components present in WCOs must be met for polymer synthesis [27,55]. A very interesting work has been published by Mannu and coworkers which reports an extensive characterization of different WCOs subjected to several different frying cycles and purified with different methods (pH, Temperature) [46]. This complex study was carried out to increase the standardization of pretreatment procedures, improving WCOs’ recyclability. In fact, according to the authors, the combination of data acquired allowed the determination of the best recycling protocols for a given WCO [45,46].

Leaving aside its main use for biodiesel production, WCOs can be chemically modified to produce bio lubricants [48,114], bio surfactants [110,115], bio-based plasticizers [116,117,118], polymers and polymer additives [55,57,119,120], binders and additives for roads and constructions [51,121,122,123,124], and bio solvents [31]. All of these applications will be further discussed in separate sections.

## 7. WCOs for Bio Lubricants and Bio Surfactants

### 7.1. Bio Lubricants

**Figure 4:**
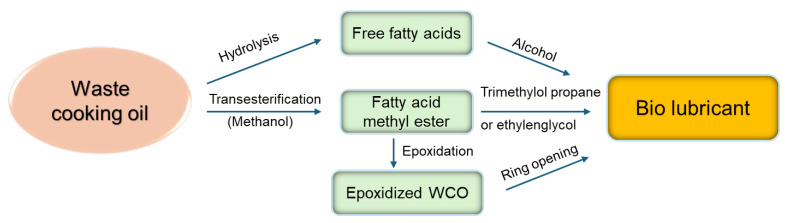
Main chemical reactions to produce bio lubricants from WCOs.

Bio lubricants (BLs) are anti-friction agents with improved viscosity, higher thermal tolerance, and lower volatility compared to petroleum-based lubricants. The BL world market had a value of 2 Mio$ in 2022 and is projected to reach a value of 2.8 Mio$ by 2031, registering a CAGR of 3.8% during the forecast period of 2023–2031 [125]. WCOs are an efficient and environmentally sustainable feedstock for BLs due to their high lubricity, high biodegradability, low volatility, and low cost. Different modification routes have been reported for the conversion of WCOs into BLs, such as hydrolysis, esterification, transesterification, hydrogenation, and epoxidation, using both chemical and enzymatic catalyzed reactions [114,126,127,128,129,130], as summarized in Figure 4.

The different routes foresee the hydrolysis of WCOs to FFAs, followed by esterification with high-molecular-weight alcohols or polyols [131,132,133], or the transesterification of WCOs to FAMEs, followed by transesterification with high-molecular-weight alcohols such as trimethylol propane (TMP) [134,135,136,137,138,139], or other polyols [114,140]. Alternatively, FAMEs may be epoxidized with hydrogen peroxide and acetic acid [131,141,142], followed by epoxy ring-opening by alcoholysis or hydrolysis in the presence of a catalyst [136,138,143,144].

From an industrial point of view, transesterification is one of the most feasible routes for the conversion of WCOs into BLs, and, consequently, the most investigated [114,138,145,146,147]. Although, in most cases, high conversions are reported, nevertheless, BLs produced by transesterification reactions have low viscosity indexes and modest performances at low temperatures.

Zhang et al. improved the efficiency of the process [130] by employing lipase and [HMIm][PF_6_], a recyclable ionic liquid used as catalyst, producing a product with excellent lubricant properties, such as a low pour point (−61 °C), high viscosity index (149), and high thermal-oxidation stability (312 °C). Sarno and coworkers adopted a similar transesterification process, starting with WCOs and neopentyl glycol, to produce bio lubricants [148]. A lipase immobilized on nanoparticles of modified magnetite was employed as a catalyst, showing high activity and recyclability up to ten times.

Alternatively, Jahromi and coworkers also explored the improved thermophysical and rheological properties of BLs generated by epoxidation in the presence of cyclic oxygenated compounds (cyclopentanone, cyclopentanol, anisole, and 2-methylfuran) via a four-step pathway: hydrolysis, dehydration/ketonization, Friedel−Crafts acylation/alkylation, and hydrotreatment [143]. The process allowed the achievement of BLs with a low pour point (−12 °C), a kinematic viscosity of 47.5 cP (at 40 °C), a viscosity index of 186, and a Noack volatility of 17 wt %, comparable to commercially available lubricants.

### 7.2. Bio Surfactants

Another interesting application of WCOs is to produce bio surfactants (BSs), which are employed by the food, cosmetic, textile, health, pharmaceutical, mining, and paper industries [103,115,149,150,151,152,153,154,155]. The market of BSs has grown constantly during the last two decades, and is expected to reach 4.8 Mio€ by 2025 [156].

BSs are secondary metabolites, generated by a variety of microorganisms such as fungi (yeasts) or bacteria (actinomycetes), that can either remain attached to the cell surface or be secreted outside the cell [157,158]. Like BLs, BSs also have several advantages over their synthetic equivalents, since they can be produced by fermentation from renewable feedstocks, are environmentally compatible, and show better foaming properties under a wide range of conditions (pH, salinity, and temperature) [34,159].

BSs have been classified either by their molecular weight (high and low molecular weight), ionic charges (anionic, cationic, non-ionic, and neutral), or secretion type (intracellular, extracellular, and adhered to microbial cells). However, their identification based on chemical structure remains the most widely adopted method, dividing them into glycolipids, lipopeptides, fatty acids, phospholipids, neutral lipids, and polymeric surfactants [152]. Glycolipids are low-molecular-weight amphiphiles that consist of a hydrophilic polysaccharide headgroup and one or more hydrophobic fatty acid tails [156,160], which may be produced from WCOs [161,162,163]. In fact, WCOs are an interesting feedstock for BSs, which may be converted by various yeast species into sophorolipids, rhamnolipids, mannosylerythritol lipids, and other glycolipids [110,161,162]. The use of several microbials, such as Bacillus, Pseudomonas, Acinetobacter, and Candida, has been extensively reported in the context of bio surfactant production with different methodologies (metagenomics, metatranscriptomics, and metaproteomics) [161]. Nevertheless, to date, BSs are not widely employed, due to unsustainable production costs [103].

The chemical modification of WCOs for BS production could be a more economically sustainable alternative, and, therefore, has been extensively studied. For example, Yusuff and coworkers recently reported the use of WCOs as a low-cost carbon source for producing anionic surfactant fatty acid methyl ester sulfonate via a transesterification–sulfonation process [164]. Furthermore, Permadani developed a bio-detergent from methyl ester-sulfonate (MES) produced from WCOs, using titanium dioxide nanoparticles as catalyst [165]. In fact, several studies have been reported in the literature on the production of MES from WCOs [103,166], and these are among the leading renewable surfactants and detergents at the industrial scale, exhibiting superior detergency efficiency compared to fossil-based products, lower toxicity, and better skin compatibility [108,165]. The production of MES from WCOs foresees preliminary purification, followed by transesterification to form the corresponding methyl esters, which are then reacted with sodium bisulfite as a sulfonating agent [108]. Various papers dealing with the environmental performance of BSs using WCOs as feedstock clearly demonstrate that their use significantly reduces environmental impacts compared to the use of other feedstocks [161,162,167].

In the field of branched non-ionic surfactants, Dong and coworker synthesized a new series of bio-based, branched non-ionic surfactants (ethoxylated dihydroxy stearic acid methyl ester, DMOE) from renewable oleic acid derived from WCOs [168]. The results showed that DMOE has low foaming ability, strong defoaming ability, good wetting performance, and outstanding emulsifying capabilities, enabling the application of these surfactants for industrial cleaning activities.

Another interesting study has been published by Khalaf and coworkers [169] on the preparation and use of cationic gemini surfactants prepared from WCOs as green inhibitors against acidic corrosion of N80-steel. Gemini surfactants are also highly demanded worldwide for their promising applications in the paper, medical hygiene, and textile industries [170]. Additionally, cost-effective and efficient adsorbent macro-porous organic polymers based on WCOs were synthesized on a Kgs scale by Wu and coworkers, which were able to remove oil and heavy metals from wastewater (≥94%), exhibiting excellent hydrophobic and super-oleophilic properties [122].

## 8. WCOs for Polymer Additives

**Figure 5:**
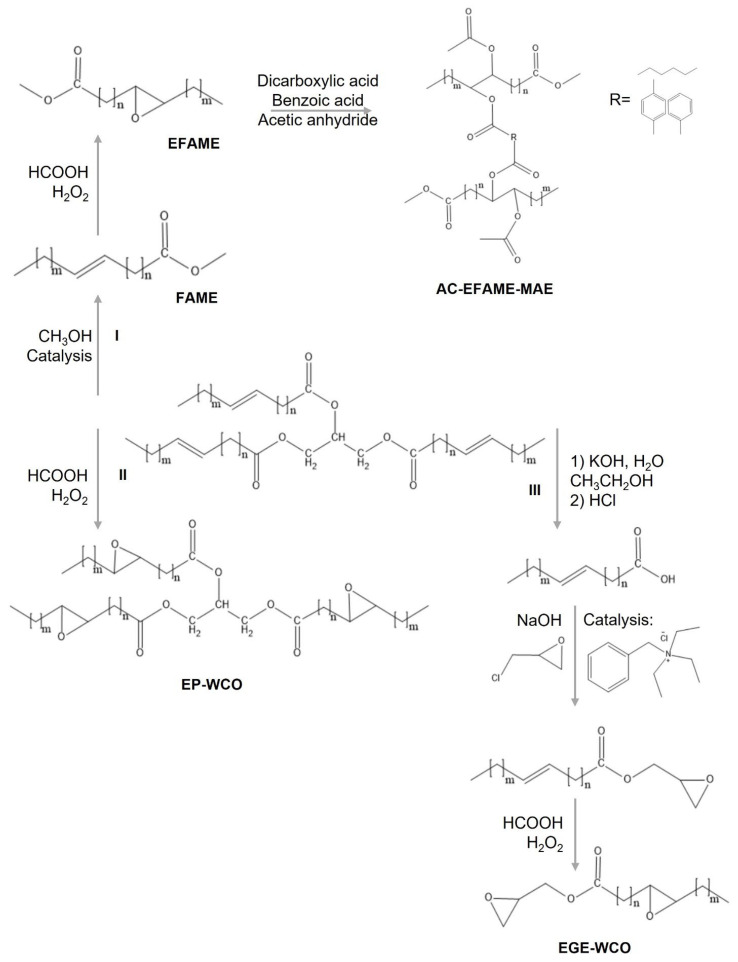
Examples of PVC additives prepared from WCOs.

With the introduction of new regulations that are increasingly restrictive, on the use of components classified as toxic, research is opening up new horizons for the use of new bio-based components as additives in the plastics industry [31,116]. The addition of plasticizers in the formulation of polymers plays a very important role in modifying the chemical and physical properties of plastic manufacts [31,171,172]. Plasticizers have been extensively used to produce flexible polyvinyl chloride (PVC) plastics, which otherwise are hard and brittle [173,174,175]. Phthalate esters, conventionally employed at the industrial level for PVC production, are known to exhibit a migration phenomenon from the polymer matrix to the surrounding media and to accumulate in the environment [116,171,175,176,177], and they are subject to REACH restriction. Due to their negative impact on human health and the environment, they have been banned in several countries in the production of toys and packaging materials [118,178,179]. In this context, the syntheses of epoxidized soybean oil (ESO), methyl epoxy soyate, amyl epoxy soyate, acetylated derivatives of castor oil, tall-oil fatty esters, and dicapryl sebacate have been described as alternative ecofriendly substitutes for phthalate esters, yet with limited applications, due to the high costs of the starting materials used [173,177,178]. More sustainable alternatives have recently been reported which employ WCOs as feedstock [117,118,179,180,181,182]. As reported by Liu et al. [183], acetylated-fatty acid methyl ester-malic acid ester (AC-FAME-MAE), obtained by modification of WCOs (Figure 5, route I), may be used as additive in PVC formulations, giving a product with chemical and physical performances that are in line with those of the common industrial additive (phthalate), making it an excellent environmentally safe substitute for phthalate esters. Cai and coworkers also reported the synthesis of bio-plasticizers from WCOs according to the strategy reported in Figure 5, routes II and III [182]. Two different highly epoxidized compounds (EGE-WCO and EP-WCO) were obtained, and their characteristics as plasticizers for PVC were tested.

EGE-WCO with an epoxy value of 6.57% showed high compatibility with PVC and high plasticizing efficiency, decreasing the Tg of PVC films from 62.8 °C to 15.8 °C and improving their elongation at break. Migration phenomena of EGE-WCO were found to be negligible. Additionally, Jia and coworkers proposed the synthesis of a covalently bonded plasticizer to the PVC backbone (WCOs methyl esters), avoiding possible migration phenomena, which have also been reported with some WCO-based plasticizers [116].

## 9. WCOs for Polymer Synthesis

### 9.1. Polyurethane

Polyurethanes (PUs) are an important class of polymers that had a global market of about 26 Miot in 2022, which is estimated to reach 31 Miot by 2030, positioning PUs as a major player in the polymer market [45,184,185,186]. In fact, due to the broad chemical and physical characteristics of these polymers, it is possible to produce flexible and rigid foams, or nonporous materials for different applications, ranging from the automotive sector to the construction industry [187,188]. Currently, PUs are prepared from virgin naphtha; however, scientific research is pushing towards replacement with renewable raw materials. In this context, many efforts have been made to prepare bio-based polyols from WCOs and achieve more renewable and sustainable PUs [57,189,190,191,192].

**Figure 6:**
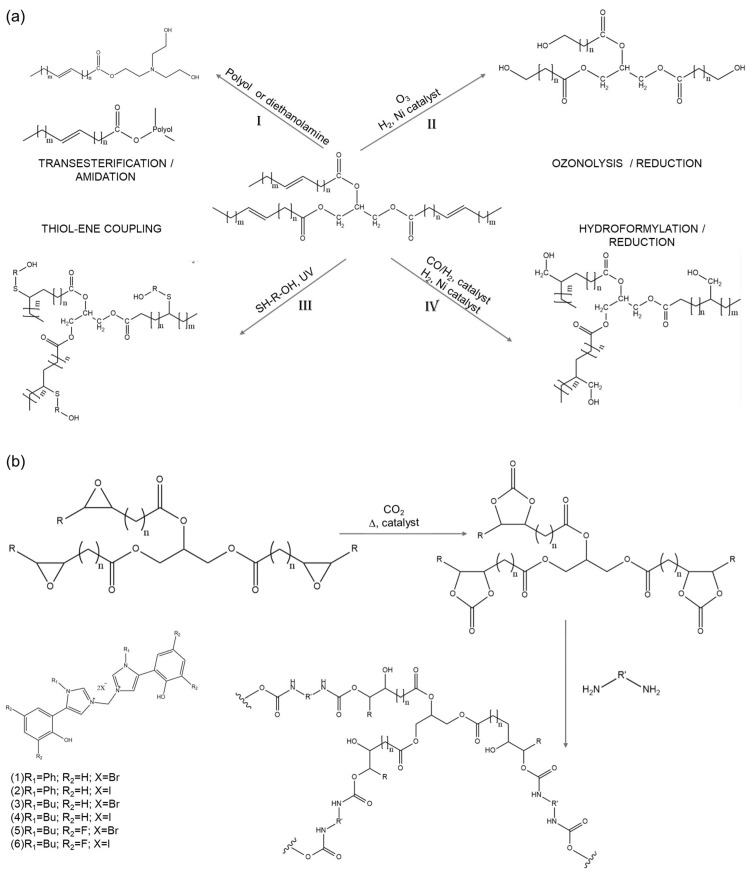
Different strategies for the synthesis of polyols from vegetable oils and WCOs.

Although many strategies have been used to produce polyols from vegetable oils, such as transesterification, epoxidation, ozonolysis, thiolene coupling reactions, hydroformylation, and photochemical oxidation (Figure 6) [187,193,194,195,196], polyols from WCOs are mainly obtained by transesterification or epoxidation (Figure 6a, route I and Figure 6b) [186,191,197,198].

Polyols generated from the transesterification of WCOs are mixtures of triglycerides, monoglycerides184- and diglycerides, together with glycerol (Figure 6a, route I). Since transesterification is an equilibrium reaction, to achieve high ester yields, an excess of alcohol, in most cases methanol, must be used to shift the equilibrium towards the products. A catalyst (alkali, acid, or enzyme) is also added to improve the reaction rate and ester yields [199,200,201].

Kuranska and coworkers reported the synthesis of polyols from WCOs in the presence of ethylene glycol, propylene glycol, diethylene glycol, and triethanolamine (Figure 6a, route I), and their characterization by Gel Permeation Chromatography [198,202]. Taking into consideration the principles of Green Chemistry, the work by Kuranska and coworkers tried to maximize the incorporation of all reagents used in the final product, avoiding solvents and employing renewable reagents, so that bio polyols produced under optimized reaction conditions could be used, without purification, for PU synthesis [202]. A different approach has been reported by various researchers, who have reported the modification of WCOs by epoxidation and ring-opening reactions, allowing them to obtain rigid polyurethane foam with chemical and physical properties like those of common polyurethane materials used in building and construction [197,203,204]. Particular attention has been given to the optimization of the catalysts employed for epoxidation and ring-opening reactions, and the data reported demonstrate that Amberlite IR-120, an ion-exchange resin, and tetrafluoro boric acid give the highest yields and selectivities [203,204]. Interestingly, it has been demonstrated that modifying the additives used to produce PUs containing WCO-based polyols enables the adjustment of their physical–mechanical properties and biodegradability [204]. For example, the combination of polyethylene glycol (PEG), 4,4′-diphenylmethane diisocyanate (MDI), and WCOs allows the achievement of biodegradable PU sheets.

Salleh and colleagues [191] reported the preparation with waste cooking oils (WCOs) of polyurethanes (PUs) that exhibited suitable properties for solid polymer electrolyte production. This was achieved through pre-polymerization (epoxidation and hydrolysis) in the presence of lithium iodide, as a charge carrier, and ethylene carbonate. Finally, MDI was added, and films prepared by casting technology [191,205,206]. Few other works have been published on the production of composite materials containing PUs prepared from WCO-derived polyols. For instance, Lubis and coworkers [207] tested the preparation of reinforced sugar palm fibers for the preparation of PU foams from WCOs, polyols, and toluene diisocyanate. According to the authors, WCOs promote cross-linking and improve the interfacial adhesion between the fiber and the matrix. Additionally, WCOs have been employed as precursors for super-hydrophobic coating applications through a process of amidation of WCOs, followed by functionalization with diisocyanates and fatty acids [208], or with diisocyanates and amino-terminated polydimethylsiloxane [182]. Silva and coworkers recently reported a comparative life cycle analysis of fossil and bio-based polyurethane foams, evidencing the different benefits and criticalities offered by different methods of PU production [209].

A major concern remains regarding the use of toxic diisocyanates, which are also employed to produce PUs with bio-based polyols. Alternatively, non-isocyanate polyurethanes (NIPUs) have been widely studied, yet their industrial application is still hampered by the low physical–chemical characteristics of NIPUs, together with their unsustainable production costs. Nevertheless, NIPUs constitute the most promising frontier for producing environmentally sustainable PUs, and should therefore be implemented.

Within this panorama, the efficient synthesis of polyurethanes from WCOs and CO_2_, in the presence of different organocatalysts, is a promising alternative (Figure 6b) [210,211]. This interesting alternative not only promotes the recovery and reuse of a waste (WCOs), but also of CO_2_, consequently reducing greenhouse gas emissions, and avoiding the use of fossil-based chemicals and toxic diisocyanates. Werlinger and coworkers reported the synthesis of bis-imidazole salt catalysts (Figure 6b), bearing two -OH groups, for the synthesis of cyclic carbonates derived from WCOs, employed as starting materials for the synthesis of different NIPUs by polyaddition reactions of carbonated vegetable oils with a variety of diamines. The catalysts and cyclic carbonates prepared were characterized by NMR, whereas NIPUs were characterized by NMR, IR, and GPC, and their thermal properties were studied using TGA and DSC analysis.

### 9.2. Acrylic Polymers

A wide range of applications exist for the use of vegetable oil triglycerides to prepare acrylic polymers, and similar protocols have also been adopted using WCOs as a starting point [55,212]. For example, Wu and coworkers [213,214] reported that they successfully converted WCOs from McDonald’s restaurants into acrylic polymers for use in additive 3D printing manufacturing. Filtered WCOs were reacted with acrylic acid, in the presence of boron trifluoride etherate (BF_3_xEt_2_O), at 80 °C for 4 h. The product was characterized, showing that the double bonds of the triglyceride molecule break because of the grafting of the acrylic groups. After purification to eliminate residual unreacted acrylic acid, the product could be 3D printed, either with or without the addition of a photo inhibitor. Interestingly, the polymers were found to be biodegradable, showing 18% weight loss after 14 days of incubation in soil [215]. Further papers, such as those by Yan Liu, have also studied 4D printing of multifunctional photocurable resin based on waste cooking oil [138].

Onn and coworkers recently reported the modification of WCOs through enzymatic acidolysis, carried out to increase the unsaturation sites and chemical reactivity of WCOs, in order to produce an acrylic pre-polymer which was photo-cross-linked in the presence of a photo initiator [212].

### 9.3. Alkyd Esters

**Figure 7:**
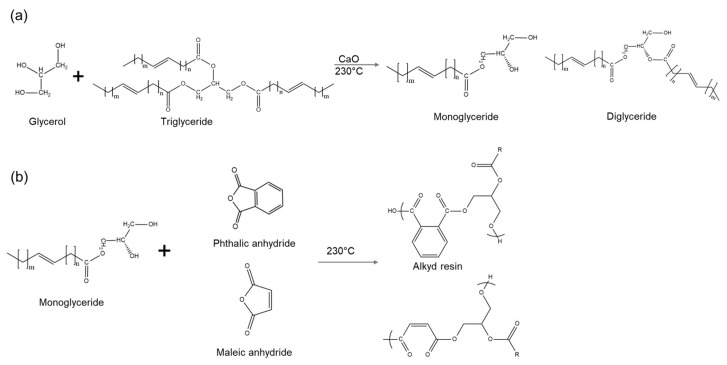
Monoglyceride process for synthesis of alkyd resins by (**a**) base or acid catalyst alcoholysis or (**b**) polycondensation reaction.

Alkyd ester resins are prepared by a condensation reaction between a polyol, a polybasic acid, and a fatty acid. Alkyd esters are used in paints, varnishes, and casting molds, with a yearly world production of around 200,000 tons [216]. Fatty acids employed for the formulation of alkyd resins may derive from vegetable oils, animal fats, or WCOs, improving their sustainability. A common strategy employed to produce alkyd resins from vegetable oils, known as the monoglyceride process, is reported in Figure 7 [55], and foresees a two-step strategy: (a) base or acid catalyst alcoholysis of WCOs, to generate monoglycerides and diglycerides; (b) a polycondensation reaction of the monoglycerides with an anhydride (aromatic or aliphatic). According to the type and concentration of anhydride used, as well as the length of the oil, the properties of alkyd ester resins can be changed.

Research carried out by Silvianti and coworkers [126] reports the synthesis of alkyd ester resins from waste palm cooking oil, using zeolites as water absorbents, in alternative to distillation. At the same time, this reduces the concentration of FFA content, contaminants, toxic products, bad odors, and color. WCOs have also been employed by Phunphoem and coworkers to produce alkyd-based printing inks for vehicles (Phunphoem et al., 2020).

### 9.4. Epoxy Resins

Epoxy resins are thermosetting polymers made of monomers that have a three-membered epoxide ring. Epoxy resins are widely used in many applications thanks to their outstanding thermal and mechanical properties, and worldwide consumption of epoxy thermoset resins is expected to reach 17.0 Mio$ by 2028 [217]. The epoxy resin industry is presently based on diglycidyl ether of bisphenol A (DGEBA), produced by a reaction between bisphenol A and epichlorohydrin. All of these chemicals are listed as highly toxic to human health; DGEBA is identified as an endocrine disruptor and a teratogen, and has long-term effects on aquatic life, and has therefore been banned from materials used in baby milk bottles [218]. Bio epoxy resins thus appear to be a very important bio-based, renewable, biodegradable, and biocompatible alternative to petroleum-based epoxy resins and triglycerides coming from WCOs, and have been demonstrated to have a wide scope of applicability [219,220]. Epoxide resins are produced either by chain-growth ring-opening polymerization or step-growth polymerization [220]. Fernandes and coworkers are claimed to be the first to have used epoxy resins from WCOs, in combination with recycled carbon fibers, followed by casting, to produce composites with interesting tensile strength (53 MPa) and Young’s Modulus (3.2 GPa), and to reduce the characteristic brittleness of DGEBA [221].

## 10. Waste Cooking Oils for Roads and Constructions

The increasing need for renewable resources as alternatives to fossil-based ones has also pushed many researchers to study the use of WCOs in the construction industry. Due to their similar elemental composition to petroleum, WCOs have been widely employed for the production of construction materials, mainly for asphalt and concrete production, as described below [137,214,222,223,224,225,226,227,228,229,230,231,232,233].

### 10.1. WCOs for Asphalt Production

Many different works have been published in the last decade on the use of WCOs as additives for asphalt concrete and asphalt cement to improve various characteristics, such as softening point, viscosity, complex modulus, creep stiffness, resistance to deformation, voids in mineral aggregate and elastic recovery [73,187,234,235,236]. On the other hand, in most cases, WCO additives adversely affect penetration, ductility, phase angle, stress relaxation, and thermal cracking resistance, and reduce the overall stability of asphalt concrete [224,234,235].

Wang and coworkers investigated the use of WCOs as modifiers for fossil-based asphalt binders. Based on infrared spectroscopy, frequency sweep rheological analysis, a multiple stress creep recovery test, and linear amplitude sweep tests, the authors verified that WCO-modified binders displayed a higher carbonyl index, lower binder stiffness and softening effects, and reduced rutting resistance at high temperatures [52]. Also, the fatigue life of bio binders under cyclic fatigue loading was significantly improved by increasing the wt% of WCOs by wt of bitumen, further confirming their potential applicability as sustainable asphalt binders [52].

Additionally, WCOs have been extensively studied for the recycling of reclaimed asphalt pavement (RAP) and grinded tire rubber (GTR) that would otherwise be discarded [98,230,236]. The recycling of RAP generates economic savings, reducing the exploitation of non-renewable resources, energy consumption, and polluting emissions in mining and transportation operations [237,238]. However, as bitumen ages, it loses its original properties, causing distresses in pavements that may endanger traffic safety and reduce travel comfort. In fact, when quantities above 20 wt.% of RAP by wt. of bitumen are used, a gradual decrease in fatigue cracking and low-temperature cracking of pavements is observed [239]. WCOs are an economically and environmentally sustainable alternative to fossil-based rejuvenating agents, allowing these shortcomings to be solved [236]. Independently from the geographical origin of RAP and WCOs, data reported in the literature generally agree that WCOs between 1 and 8% wt.% by weight of bitumen positively affect penetration value [234,239,240,241,242,243,244]. This can be attributed to the alteration in the consistency of asphalt, contributing to improved fluidity and workability of the modified asphalt.

Pretreatment of WCOs is normally required to reduce acidity, due to variable quantities of FFAs present, which negatively influence performance [51]. Common pretreatment practices are esterification, transesterification in the presence of methanol, or refining processes which promote the overall performance of WCO-modified bitumen [245,246,247]. An interesting study has recently been published by Bardella and coworkers on the possibility of achieving highly performing rejuvenating agents by chemical modification of WCOs, or hydrolysis of WCOs by transesterification (other than methanol) or amidation reactions, to achieve various WCO esters and amides. All samples were characterized according to their nuclear magnetic resonance, melting point, and boiling point. The efficiency of WCO esters was assessed by means of the Asphaltenes Dispersant Test and the Heithaus Parameter, showing that bitumen blends containing 25 wt.% of WCOs by weight of bitumen, modified with 2-phenylethyl alcohol, performed better than bitumen alone [51].

Alternatively, in a recent work, Enfrin and coworkers studied the pretreatment of WCOs by the epoxidation reaction, which were used as rejuvenating agents for RAP [248]. This low-cost procedure allowed rejuvenating agents to be produced with immediate cracking resistance comparable to that of commercially available fossil-based rejuvenating agents, and higher long-term cracking resistance. The authors suppose that epoxide groups promote dispersion of the asphaltene clusters of bitumen, creating bridges between the molecules and preventing their agglomeration, slowing aging phenomena.

Another interesting work has been reported on the rejuvenating and self-healing efficiency of WCO capsule preparation by mixing WCOs with sodium alginate or chitosan powder, which were then poured in a CaCl_2_ solution, and capsules produced with a micro-peristaltic pump. In general, the WCO performances were superior to those of commercially available products, and, in particular, the fatigue healing performances of asphalt mixtures were improved by 10∼30% [223].

From an environmental point of view, WCO rejuvenating agents reduce greenhouse gas emissions and energy consumption. For instance, emissions of CO_2_ are reduced from 20.06 to 18.44 KgCO_2_e/t by switching from conventional bitumen to bio-bitumen, respectively, and energy consumption is reduced from 48.33 × 109 to 42.81 × 109 J per ton of material produced [130,249].

Elahi and coworkers very recently reported a study on the modification of WCOs with styrene-butadiene-styrene (SBS) from GTR to give a rubbery-solid used as a bitumen additive. WCOs promote the swelling and cross-linking process of SBS, improving the adhesion between the modified WCOs and the bitumen [238]. The following three-step process was used for the modification of WCOs: (i) filtration; (ii) pre-swelling of SBS with various wt./wt.% of WCOs/SBS (45:55 and 50:50 wt./wt.%) at 160 °C; (iii) reduction in size of the modified WCO mixtures and mixing of them with bitumen (between 5 wt.% and 25 wt.% by weight of bitumen). The data reported showed that increasing the content of SBS-modified WCOs improved the bitumen’s stiffening, temperature susceptibility, viscoelastic response, and elasticity. Also, ethylene-vinyl acetate (EVA) has been used in combination with WCOs, producing highly performing rejuvenating agents [54,249].

### 10.2. WCOs for Concrete

WCOs have also been studied for the production of sustainable cements. Li and coworkers developed a cement clinker and gypsum mixture containing different WCOs, and verified their physical mechanical characteristics, together with their economic and environmental impacts [142]. Results show that, overall, WCOs favorably improved cement grinding, and when highly unsaturated WCOs were employed, cement strength was also improved. Additionally, WCOs improved microstructure density, hardening the cement paste. The authors concluded that the use of WCOs as grinding promoters in cement processing is economically and environmentally beneficial. Recently, Liu and coworkers studied the use of WCO emulsions as shrinkage-reducing admixtures to improve concrete durability. The prepared oil and water emulsions were able to reduce cement shrinkage, enhancing its dispersion and stability [250]. The addition of different contents of WCOs and water emulsions reduced the total porosity and refined the pore size by significantly reducing Ca(OH)_2_ crystals, probably because of the saponification reaction between WCOs and Ca(OH)_2_.

## 11. WCOs as Bio Solvents

Another interesting characteristic of WCOs is their ability to capture volatile organic compounds (VOCs) [31,60] originating from different manufacturing procedures, such as emissions from rice husk pyrolysis and rubber vulcanization [60,251].

Lhuissier and coworkers [60] developed a non-aqueous phase absorption and degradation bioreactor for Volatile Organic Compounds (VOCs). The VOCs targeted were n-heptane, ethyl acetate, isopropanol, methyl isobutyl ketone, toluene, m-xylene, and 1,3,5-trimethylbenzene. The data reported showed that WCOs have an absorption efficiency which is comparable to that of fossil-based oils. Tarnpradab and coworkers employed WCOs to reduce the emissions produced during rice husk pyrolysis [251], and in this case, WCOs were able to reduce the content of the organic hydrocarbons formed. Other different examples exist for the use of WCOs for the adsorption of VOCs produced by the leather industry [252], or of other industrial contaminants such as mercury [61].

In addition, WCOs have been employed as solvents for the pretreatment of GTR, to compensate for the insufficient storage stability of GTR-modified asphalt [224,253]. GTR is one of the most common bitumen modifiers used to improve bitumen’s rheological properties. However, bitumen containing GTR generally has poor storage stability and workability, affecting the performance of the pavement [224,253]. Pretreatment of GTR with WCOs reduces polymer segregation phenomena, improving aging resistance and reducing environmental impacts [254,255]. In addition, recent research also indicates that a rejuvenator with excellent storage stability and regeneration effects can be obtained when GTR is pyrolyzed in a WCO-rich phase [256].

## 12. Other Applications

Although data are available from the literature on the use of edible [257,258] and non-edible waste oils [259,260] as organic phase change materials for food preservation, asphalt, or building thermoregulation, the use of WCOs for this scope is very limited [261,262,263,264,265,266].

Different works have reported on the use of filtered WCOs for thermal energy storage, showing they significantly reduce energy consumption compared to analogous products prepared from other virgin or waste oils [260,262].

Alternatively, phase change capsules (PCCs) have been prepared by mixing WCOs in the presence of sodium alginate and Tween80, an emulsifying agent, and microcapsules have been prepared by microfluidic technology [261]. PCCs have been employed for the preparation of foamed concrete with mechanical properties that are within standard requirements (strength > 4.80 MPa) and interesting thermal insulation properties.

## 13. Conclusions

Based on literature data reported in this work, it evidently emerges that WCOs are a valuable type of food waste that can be used as feedstock to produce a variety of different bio-based polymeric materials. Although WCOs are renewable starting materials for sustainable biorefinery, nonetheless, they struggle to find industrial applications. The complexity and variability in the composition of WCOs, together with an inadequate collecting system, are among the main barriers for the introduction of WCOs as feedstock at the industrial level. Competition for the use of WCOs in the biofuel market is also a drawback, reducing their availability. The large-scale adoption of WCO-derived polymers faces significant economic challenges, despite their environmental advantages and coherence with circular economy principles. These challenges include the following:

1. Feedstock availability and cost variability: WCO supply is inconsistent due to regional and seasonal variations, high transportation costs, limited collection infrastructure, and competition from industries like biodiesel production, making it an expensive feedstock.
2. High initial capital investment: scaling production requires costly infrastructure and advanced technologies.
3. Processing costs and technological barriers: converting WCOs into polymers is more expensive than traditional methods, due to the need for impurity removal, quality control, and, often, complex processing technologies.
4. Market competition and price sensitivity: competing with low-cost petrochemical polymers is difficult without incentives, especially in price-sensitive sectors.
5. Regulatory and certification challenges: complying with varying regional regulations and obtaining the necessary certifications adds significant financial and administrative burdens.
6. Consumer perception and demand uncertainty: limited awareness and misconceptions about waste-derived materials hinder market demand, requiring costly consumer education campaigns.
7. Competition for alternative uses of WCOs: consolidated applications like biodiesel production, supported by mature markets and incentives, reduce the availability of WCOs for polymer production.
8. Limited policy support and economic incentives make it difficult for WCO-based polymers to compete with conventional materials.

To overcome these problems, future efforts should address the adoption of legislative strategies aiming to harmonize the collecting system throughout the EU and promote campaigns to raise awareness among consumers of the importance of WCOs’ separate collection. Furthermore, strengthening WCO collection networks will help to enhance their availability and reduce costs. Additionally, investments in research to develop cost-effective and scalable technologies are necessary, as well as supportive policies, including subsidies and tax incentives, to promote the upcycling of WCOs on an industrial scale.

From a technological point of view, many different strategies and applications exist to produce high-value polymeric products from WCOs, although only a few of them have been implemented at the industrial level today. This evidently testifies that further efforts need to be made both by the scientific community and the industry to reduce the complexity of the processes necessary to transform WCOs into polymeric materials, which are strictly connected to collecting systems and management capacities. Once WCOs are collected, managed, and used properly, they will surely become a valuable feedstock for the polymer industry, satisfying most of the sustainable development goals of the 2030 agenda and of the principles of green chemistry. Further industrial prospects, the market availability of WCO-based polymers, and patent analysis are under investigation, and will be the object of the next publication.

## Figures and Tables

**Table 1:** Chemical physical characteristics of vegetable oils and WCOs derived.

Component	Sunflower Oil ^a^	Sunflower Oil WCO ^b^	Rapeseed Oil ^a^	Rapeseed Oil WCO ^a^	Palm Oil ^a^	Palm oil WCO ^a^	Sun Foil ^a^	Sun Foil WCO ^a^
Saturated fatty acids	71.5	32.0				80.0–93.0	74.4	73–15
Monounsaturated fatty acids	-	62.0				20.0–7.0		27–6
Polyunsaturated fatty acids	28.5	6.0					25.6	0–79
Acidic value (mg KOH/g oil)	0.30	2.29	0.06	1.06		0.66–1.13		0.72–1.44
pH	7.38	5.34			6.34	5.73–6.19	8.63	6.14–6.61
Density at 20 °C (kg/m^3^)	919.21	920.40	918.00	929.00	919.48	923.2–913.4	919.6	919.8–923.2
Kinetic viscosity at 40 °C (mm^2^/s)	28.744	31.381	63.286	68.568	27.962	44.254–38.407	28.224	43.521–35.236
Molecular weight (g/mol)	670.82	51.94	869.16	871.01	535.08	135.66–586.05	119.71	55.18–395.28

^a^ Ref. [13]. ^b^ Ref. [111].

## Data Availability

The original contributions presented in this study are included in the article. Further material is available on request.
